# The effect of care transition pathway implementation on patients undergoing joint replacement during the COVID-19 pandemic: a quasi-experimental study from a tertiary care hospital orthopedic department in Beijing, China

**DOI:** 10.1186/s13018-021-02511-5

**Published:** 2021-06-01

**Authors:** Ya-ping Xu, Pei-yu Zhao, Yi-tong Bai, Shuang Li

**Affiliations:** 1 Department of Orthopedics, China-Japan Friendship Hospital, Beijing, China; 2 Bone Necrosis and Joint Preservation Reconstruction Center, China-Japan Friendship Hospital, Beijing, China; 3 Department of Bone and Joint Surgery, 2 Yinghua Dongjie, Hepingli, Chaoyang District, Beijing, 100029 China; 4 Department of Nursing, China-Japan Friendship Hospital, Beijing, China

**Keywords:** Care transition, Quality, Quality improvement, Joint replacement, COVID-19

## Abstract

**Background:**

The coronavirus disease (COVID-19) pandemic has had a massive impact on individuals globally. The Chinese government has formulated effective response measures, and medical personnel have been actively responding to challenges associated with the epidemic prevention and control strategies. This study aimed to evaluate the effect of the implementation of a care transition pathway on patients that underwent joint replacement during the COVID-19 pandemic.

**Methods:**

A quasi-experimental study was designed to evaluate the effect of implementing a care transition pathway for patients who underwent joint replacement during the COVID-19 pandemic in the orthopedic department of a tertiary care hospital in Beijing, China. Using a convenient sampling method, a total of 96 patients were selected. Of these, 51 patients who had undergone joint replacement in 2019 and received treatment via the routine nursing path were included in the control group. The remaining 45 patients who underwent joint replacement during the COVID-19 epidemic in 2020 and received therapy via the care transition pathway due to the implementation of epidemic prevention and control measures were included in the observation group. The quality of care transition was assessed by the Care Transition Measure (CTM), and patients were followed up 1 week after discharge.

**Results:**

The observation group was determined to have better general self-care preparation, written planning materials, doctor-patient communication, health monitoring, and quality of care transition than the control group.

**Conclusions:**

A care transition pathway was developed to provide patients with care while transitioning through periods of treatment. It improved the patient perceptions of nursing quality. The COVID-19 pandemic is a huge challenge for health professionals, but we have the ability to improve features of workflows to provide the best possible patient care.

**Supplementary Information:**

The online version contains supplementary material available at 10.1186/s13018-021-02511-5.

## Background

The coronavirus disease (COVID-19) pandemic has had a massive impact on people globally [1–3]. The Chinese government has formulated effective response measures, and medical personnel have been actively responding to challenges associated with the epidemic prevention and control strategies. In this context, as health care professionals, we must not only implement measures to prevent COVID-19 spread, but should also provide quality health care services to patients with diverse needs. As the staff of the orthopedic ward of a tertiary hospital, we are required to strictly implement hospital management regulations which strictly prohibit patients from being accompanied by their family. As an alternative, patients are permitted cloud visits in which they are allowed contact with the family online. Simultaneously, high-quality care services for patients must be provided. Influenced by the traditional Chinese culture of “filial piety,” patients have previously tended to be accompanied by relatives throughout periods of hospitalization. The sudden change in policy was a great challenge for the medical staff, patients, and their relatives. To assist the patients who underwent joint replacement to complete the perioperative care transition for restored function and transition to normal life, new guidelines were being implemented by the medical staff.

The term “health care transition” refers to “a set of actions designed to ensure the coordination and continuity of health care as patients transfer between different locations or different levels of care within the same location. Transitional care, which encompasses both the sending and the receiving aspects of the transfer, is essential for persons with complex care needs [4].” This study assessed the use of a care transition pathway for patients that underwent joint replacement during the COVID-19 pandemic, which was developed to adapt to changes associated with the newly implemented, outbreak-associated regulations.

It is worth mentioning that the “What, Why, Who, Where, When, and How (5W1H)” method was used in the development and implementation of the care transition pathway. Currently, China is progressing toward the end of the epidemic and has adopted an orderly and active approach for resuming work. As this happens, the care transition pathway we developed has the potential to be increasingly used to guide the transitional care of patients who undergo joint replacement.

## Methods

### Design and setting

The study had a quasi-experimental design. A total of 96 patients who underwent total hip or knee joint replacement at a tertiary hospital were selected as study subjects using convenience sampling. Of these, 51 patients who underwent joint replacement in 2019 and who received the routine nursing path care were included in the control group. The remaining 45 patients, who underwent joint replacement during the COVID-19 epidemic in 2020 and received treatment guided by the care transition pathway, were included in the observation group (Fig. S1). The care transition pathway was developed by a multidisciplinary team to address practical clinical problems using the 5W1H method. The application of the 5W1H methodology in the study of scientific problems enables researchers to think systematically and scientifically [5].

The Care Transition Measure (CTM) has been used to assess the effectiveness of the care transition pathway for patients with joint replacement [6] and is a valid and reliable tool used for measuring the quality of transitional care [7, 8]. The reliability and validity of the Chinese version (CTM-C) of the assessment have been established and are used to assess the quality of transitional care in mainland China [9]. The content validity index of the total scale of the CTM-C is 0.99. The test is composed of 17 items, each evaluated using a 4-point Likert scale ranging from “strongly disagree” to “strongly agree.” Factor analysis revealed four dimensions, as follows: general self-care preparation, written plan, doctor-patient communication, and health monitoring. The analysis of the four aforementioned factors revealed a cumulative variance of 58.96%. The Cronbach’s α for the total scale was 0.85, while that of each factor varied from 0.61 to 0.89 [9]. Finally, the average score of items expressed as a percentage was used as the scale score, and the following conversion formula was applied: scale score = (average score of items − 1) × 100 ÷ 3. The score ranged from 0 to 100, with higher scores indicating improved continuous in-hospital care, relative to lower scores [10].

Two volunteers with no conflicting interests were trained in the use of CTM-C. Demographic data of 96 participants and responses to the 17 CTM-C items that were collected through a follow-up telephone interview 1 week after discharge were recorded.

The study was approved by the institutional review board of China-Japan Friendship Hospital and adhered to the tenets of the Helsinki Declaration. The written informed consent of study participants was not required because the survey administered was anonymized and posed less than minimal risk.

### Data analysis

All data analyses were performed using the IBM SPSS Statistics for Windows software, version 25.0 (IBM Corp.; Armonk, NY, USA). Descriptive statistical analysis was used to summarize the demographic characteristics of study participants. Categorical data were reported as frequencies. The non-normally distributed data were reported as medians and quartiles [M (Q25, Q75)]. Comparisons were made using the Pearson chi-square and Fisher’s exact tests. The Mann-Whitney U test was implemented as a non-parametric test of the two independent samples. The differences in the four dimensions considered and the overall quality of transitional care were also analyzed using the Mann-Whitney U test. A two-tailed value of *P* < 0.01 was considered statistically significant.

## Results

### Characteristics of the respondents

Demographic characteristics of the study participants are shown in Table 1. Demographic data collected from both groups of study participants included gender, age, and highest education level, and differences between the groups were not statistically significant (*P* > 0.05).

**Table 1:** Characteristics of the respondents (*N* = 96)

Characteristic	Categories	CG (*N* = 51)	OG (*N* = 45)	χ^2^	*P* value*
Gender	Male	18	16	0.001^a^	0.979
	Female	33	29		
Age (years)	< 60	21	17	0.115^a^	0.734
	≥ 60	30	28		
Highest education level attained	Junior high school education or below	37	33	0.162^b^	1.000
	Technical secondary school/high school	11	9		
	College or above	3	3		

*CG*, control group; *OG*, observation group; *TKA*, total knee arthroplasty; *UKA*, unicompartmental knee arthroplasty; *THA*, total hip arthroplasty

^a^Pearson chi-square

^b^Fisher’s exact test

*P values were calculated using the χ^2^ method

### Comparison of the quality of transitional care provided to the two groups considered

As shown in Table 2, the observation group, for whom the care transition pathway was implemented, had higher general self-care preparation, written plan, doctor-patient communication, health monitoring, and transitional care quality scores than the control group, and the difference was statistically significant (*P* < 0.01).

**Table 2:** Comparison of CTM-C-determined joint replacement transitional care quality in patients of both groups considered

Groups	Number	Dimension 1 [M (Q25, Q75)]	Dimension 2 [M (Q25, Q75)]	Dimension 3 [M (Q25, Q75)]	Dimension 4 [M (Q25, Q75)]	Total factor [M (Q25, Q75)]
CG	51	95.83 (83.33, 100.00)	100.00 (83.33, 100.00)	100.00 (83.33, 100.00)	88.89 (55.55, 100.00)	90.20 (82.35, 98.04)
OG	45	100.00 (95.83, 100.00)	100.00 (100.0, 100.00)	100.00 (100.00, 100.00)	100.00 (83.33, 100.00)	100.00 (94.11, 100.00)
Z		−2.663	−3.039	−2.619	−3.163	−3.789
P**		0.008*	0.002*	0.009*	0.002*	0.000*

Dimension 1, general self-care preparation; dimension 2, written plan; dimension 3, doctor-patient communication; and dimension 4, health monitoring

*CTM-C*, Chinese version of Care Transition Measure; *M*, median; *Q*, quartile; *CG*, control group; *OG*, observation group

*Significant difference among the groups (*P* < 0.01)

**P values were calculated using the Mann-Whitney U test

## Discussion

### Clinical practice challenges during the COVID-19 pandemic

For health care leaders, the COVID-19 pandemic has been extremely disruptive and has caused challenges that required rapid, dynamic, and innovative responses [11–14]. In China, through our efforts, we have overcome the most difficult period of the COVID-19 pandemic and are now in the process of resuming production in an orderly manner. In the daily management of inpatients, in order to effectively prevent the occurrence of COVID-19-related infections in the hospital, members of medical institutions proposed a zero accompaniment and zero visitation strategy, which played an important role in the prevention and control of the epidemic in China. In the meantime, the challenges in clinical practice are something that health care workers have to think about and respond to carefully. In the past, due to the influence of the culture of filial piety, patients were often accompanied by their close relatives throughout hospitalization. In general, practitioners were accustomed to family member involvement in patient care. Health care professionals work with family members to help patients with joint replacement recover, and successfully complete the transition from the hospital to the home environment. The prohibition of visitors has changed this transition. Experience has shown that it is necessary to deal with patients with literacy problems, memory loss, severe cognitive medical knowledge deficiencies, insufficient learning ability regarding functional exercise methods, and so on. Further, medical staff must guide clinical practice based on the expert consensus regarding optimal recovery after total hip and knee arthroplasty in China [15–17]. Under current conditions, a clear path guiding clinical work is needed to make workflows clearer and improve the efficiency and organization of nursing care. We should not only pay attention to the experience of patients in the hospital, but also consider the interventions needed to treat the core problems of patients after discharge to provide continuous care throughout the transition from hospitalization to home. As shown in Fig. S1, the care transition pathway clearly guides the clinical work from the perspective of 5W1H.

### Effect of the care transition pathway on patients with joint replacement

This study showed that the observation group, who received treatment based on the care transition pathway, scored higher than the control group when general self-care preparation, written plan, doctor-patient communication, health monitoring, and transitional care quality were assessed, and the difference was statistically significant. Applying the care transition pathway in the treatment of patients who have undergone joint replacement has the potential to improve patient satisfaction and nursing quality during the COVID-19 pandemic. The use of care in the transition pathway in the treatment of patients who received joint replacement will continue to be beneficial throughout the implementation of the zero accompaniment and zero visitation management strategy. This is, to the best of our knowledge, the first report of Chinese clinical practitioners with experience in treating patients who received joint replacement during the COVID-19 epidemic, and the first study to investigate the effect of the care transition pathway on joint replacement outcomes during the COVID-19 pandemic. It is worth mentioning that the care transition pathway reported here has been used to treat more than 600 patients who have received joint replacement and has been well received. However, our study does have some limitations. First, participants were selected from patients who underwent joint replacement in a single orthopedic department of a level 3, class A hospital. China is a developing country with unevenly distributed medical resources. Medical institutions of different levels provide medical care of differing qualities. Our results may not be generalizable to other types of practices or medical institutions. Second, the sample size of the study was not large, which also may limit the generalizability of our results.

## Conclusion

Faced with the challenges of the COVID-19 pandemic, health care providers need to adapt to local conditions and challenges, and continuously improve their methods to improve patient experiences. Clinical care practices based on the care transition pathway, which has been used to improve treatment of joint replacement patients, can help patients smoothly transition from the hospital to home and improve the quality of transitional care. Taken together, our findings suggest that promoting the use of these practices is worthwhile.

## Supplementary Information

**Additional file 1: Fig. S1.** Care transition pathway for patients with joint replacement.

## Data Availability

Data collected from the survey were anonymized. The raw data from which the paper’s results were derived can be made available on request.

## References

[1] World Health Organization (2020). Coronavirus disease (COVID-2019) situation reports.

[2] World Health Organization WHO, director. General’s opening remarks at the media briefing on COVID-19. https://www.who.int/dg/speeches/detail/who-director-general-s-opening-remarks-at-the-media-briefing-on-covid-19%2D%2D-11-march-2020. Accessed 11 Mar 2020.

[3] Wang L, Qi N, Zhou Y, Zhang H (2020). Prevention and infection control of COVID-19 in nursing homes: experience from China. Age Ageing.

[4] Coleman EA, Boult CE (2003). American Geriatrics Society Health Care Systems Committee for the American Geriatrics Society Health Care Systems Committee. Improving the quality of transitional care for persons with complex care needs. J Am Geriatr Soc.

[5] Ma N, Liu Y (2020). Risk factors and risk level assessment: forty thousand emergencies over the past decade in China. Jamba..

[6] Coleman EA, Smith JD, Frank JC, Eilertsen TB, Thiare JN, Kramer AM (2002). Development and testing of a measure designed to assess the quality of care transitions. Int J Integr Care.

[7] Bakshi AB, Wee SL, Tay C, Wong LM, Leong IY, Merchant RA (2012). Validation of the care transition measure in multi-ethnic South-East Asia in Singapore. BMC Health Serv Res.

[8] Cao X, Chen L, Diao Y, Tian L, Liu W, Jiang X (2015). Validity and reliability of the Chinese version of the care transition measure. PLoS One.

[9] Li YH, Wang XY, Liu Y, Lu Y, Li L, Li XP (2014). Reliability and validity of Chinese version of Care Transition Measure. Chin Nurs Manag.

[10] Coleman EA, Mahoney E, Parry C (2005). Assessing the quality of preparation for posthospital care from the patient’s perspective: the care transitions measure. Med Care.

[11] Zorn CK, Pascual JM, Bosch W, Thiel DD, Francis D, Casler JD (2020). Addressing the challenge of COVID-19: one health care site’s leadership response to the pandemic. Mayo Clin Proc Innov Qual Outcomes.

[12] Luceri F, Morelli I, Accetta R, Mangiavini L, Maffulli N, Peretti GM (2020). Italy and COVID-19: the changing patient flow in an orthopedic trauma center emergency department. J Orthop Surg Res.

[13] Simon MJK, Regan WD (2021). COVID-19 pandemic effects on orthopaedic surgeons in British Columbia. J Orthop Surg Res.

[14] Soler E, Farah SN, Bustos VP, Medina SEM, Gómez JF, Lema EM, Moreno CÁ (2021). Experience of clinical screening for COVID-19 among patients undergoing elective orthopedic surgeries: an alternative proposal. J Orthop Surg Res.

[15] Xisheng W (2016). Expert consensus in enhanced recovery after total hip and knee arthroplasty in China: perioperative management. Chin J Bone Joint Surg.

[16] Shen B (2016). Weng Xs, Liao R, Qu Tb, Zhang Xl, Yan Sg, et al. Expert consensus in enhanced recovery after total hip and knee arthroplasty in China: pain and sleep management. Chin J Bone Joint Surg.

[17] Zhou ZK, Wong XS, Xiang B (2016). Expert consensus in enhanced recovery after total hip and knee arthroplasty in China: diagnosis and treatment of perioperative anemia. Chin J Bone Joint Surg.

