# Supplementary material for: The effect of care transition pathway implementation on patients undergoing joint replacement during the COVID-19 pandemic: a quasi-experimental study from a tertiary care hospital orthopedic department in Beijing, China
Source: J Orthop Surg Res. 2021 Jun 1;16:356. doi: 10.1186/s13018-021-02511-5 (PMC8167389; doi:10.1186/s13018-021-02511-5)
Supplement: Supplementary file 1 — Additional file 1: Fig. S1. Care transition pathway for patients with joint replacement. [file 13018_2021_2511_MOESM1_ESM.docx]

Fig. 1 Care transition pathway for patients with joint replacement

| When | Where | Who | What | Why | How |
| --- | --- | --- | --- | --- | --- |
| On admission | Hospital ward | Doctors and nurses | 1. Personnel introduction 2. Introduction of environmental facilities 3. Inform of hospitalization rules and regulations 4. Risk factor assessment and notification (falls, stress injury, venous thromboembolism, etc.) | 1. To quickly and optimally adjust the role of patients | 1. Oral explanation 2. Paper material display 3. Guided tour |
| Pre-operation | Hospital ward | Doctors, nurses, rehabilitators (when necessary), pharmacists (when necessary), anesthesiologists | 1. Perfecting preoperative examination and preparation 2. Make joint decisions about surgery and anesthesia, and adequate preoperative information 3. Risk factor reassessment and recommunication (falls, stress injury, venous thromboembolism, etc.) 4. Pre-guided functional exercises | 1. To prepare well before surgery, and facilitate rapid recovery after the joint replacement | 1. Oral explanation 2. Paper material display 3. Video presentation 4. Demonstration teaching and counter-demonstration 5. WeChat for information dissemination |
| Post-operation | Hospital ward | Doctors, nurses, rehabilitators (when necessary), pharmacists (when necessary), anesthesiologists | 1. Notice of matters 2. Risk factor reassessment and recommunication (falls, stress injury, venous thromboembolism, etc.) 3. Supervision and guidance for functional exercise | 1. To achieve rapid recovery and return to daily life | 1. Oral explanation 2. Paper material display 3. Video presentation 4. Demonstration teaching 5. WeChat for information dissemination |
| On discharge | Hospital ward | Doctors, nurses, rehabilitators (when necessary), pharmacists (when necessary) | 1. Insurance Particulars 2. Medication guidance 3. Self-observation and management of complications 4. Notes on Review Items 5. Establishment of contact information | 1. To reduce the chance of readmission and improve the patient's medical experience | 1. Oral explanation 2. Paper material display |
| After discharge | Home | Doctors, nurses | 1. Track status 2. Answer questions | 1. To effectively communicate | 1. Telephone follow-up |
